# Functional characterization of cotton genes responsive to *Verticillium dahliae* through bioinformatics and reverse genetics strategies

**DOI:** 10.1093/jxb/eru393

**Published:** 2014-10-17

**Authors:** Lian Xu, Wenwen Zhang, Xin He, Min Liu, Kun Zhang, Muhammad Shaban, Longqing Sun, Jiachen Zhu, Yijing Luo, Daojun Yuan, Xianlong Zhang, Longfu Zhu

**Affiliations:** National Key Laboratory of Crop Genetic Improvement, Huazhong Agricultural University, Wuhan, Hubei 430070, P. R. China

**Keywords:** *Arabidopsis*, data-mining, *Gossypium hirsutum*, innate immune response,, *Verticillium dahliae*, virus-induced gene silencing.

## Abstract

*Verticillium* wilt causes dramatic cotton yield loss in China. Although some genes or biological processes involved in the interaction between cotton and *Verticillium dahliae* have been identified, the molecular mechanism of cotton resistance to this disease is still poorly understood. The basic innate immune response for defence is somewhat conserved among plant species to defend themselves in complex environments, which makes it possible to characterize genes involved in cotton immunity based on information from model plants. With the availability of *Arabidopsis* databases, a data-mining strategy accompanied by virus-induced gene silencing (VIGS) and heterologous expression were adopted in cotton and tobacco, respectively, for global screening and gene function characterization. A total of 232 *Arabidopsis* genes putatively involved in basic innate immunity were screened as candidate genes, and bioinformatic analysis suggested a role of these genes in the immune response. In total, 38 homologous genes from cotton were singled out to characterize their response to *V. dahliae* and methyl jasmonate treatment through quantitative real-time PCR. The results revealed that 24 genes were differentially regulated by pathogen inoculation, and most of these genes responded to both *Verticillium* infection and jasmonic acid stimuli. Furthermore, the efficiency of the strategy was illustrated by the functional identification of six candidate genes via heterologous expression in tobacco or a knock-down approach using VIGS in cotton. Functional categorization of these 24 differentially expressed genes as well as functional analysis suggest that reactive oxygen species, salicylic acid- and jasmonic acid-signalling pathways are involved in the cotton disease resistance response to *V. dahliae*. Our data demonstrate how information from model plants can allow the rapid translation of information into non-model species without complete genome sequencing, via high-throughput screening and functional identification of target genes based on data-mining and VIGS.

## Introduction

Plants adapt to complex environments by defending themselves against a wide range of pathogens that have different lifestyles (Pieterse *et al.*, 2009). Two strategies are evolved to perceive attackers in plants (Jones and Dangl, 2006; Brutus and He, 2010). First, some common features of pathogens, called pathogen-associated molecular patterns (PAMPs), which include flagellin, lipopolysaccharides and chitin, are recognized by pattern-recognition receptors (or PRRs) in the plant cell membrane. Stimulation of such receptors ultimately leads to the activation of basal immune resistance against a wide range of pathogens called PAMP-triggered immunity (PTI; formerly named horizontal resistance). Furthermore, intracellular plant resistance proteins (R proteins) can detect pathogen effectors, secreted by the pathogens, resulting in effector-triggered immunity (ETI; formerly named vertical resistance; Jones and Dangl, 2006; Brutus and He, 2010). Generally, PAMPs are conserved across classes of pathogens and contribute to microbial fitness, while effectors are strain-specific and contribute to pathogen virulence. Both PTI and ETI can trigger induced defence responses against a variety of pathogens, known as systemic acquired resistance (SAR) (Mishina and Zeier, 2007). Meanwhile, the innate immune response is well conserved among plant species (Palma *et al.*, 2007).

Although ETI is qualitatively faster and stronger than PTI, both give rise to similar immune responses, including Ca^2+^ influx, an oxidative burst and mitogen-activated protein kinase (MAPK) cascade activation as an initial step, followed by activation of downstream signal transduction networks mediated by other signal systems or hormones in which salicylic acid (SA) and jasmonic acid/ethylene (JA/ET) play key roles (Boudsocq *et al.*, 2010; Dodds and Rathjen, 2010). Generally, pathogens can be divided into three types; that is, biotrophic, necrotrophic and hemi-biotrophic pathogens, according to their lifestyle. Plants employ different resistance mechanisms against the three types of pathogen. SA-mediated defence responses restrict host colonization by biotrophic pathogens, whereas the JA/ET pathway commonly controls defence against pathogens with a necrotrophic lifestyle (Pieterse *et al.*, 2009). Although these processes utilize separate signal pathways, considerable overlap among them exists in cross-communicating defence processes (Spoel *et al.*, 2003; Pieterse *et al.*, 2009).

*Verticillium* wilt caused by *Verticillium dahliae*, a widespread soil-borne fungus, is responsible for vascular wilt diseases in a wide range of host crops. The shortage of resistant germplasms in cotton (*Gossypium hirsutum*) makes *Verticillium* wilt the most serious disease to influence cotton production in China. The lifestyle of this fungus is complex. It has been reported that *V. dahliae* has a biotrophic lifestyle when initially invading plants and then switches to a necrotrophic lifestyle during the late stages of infection (Thaler *et al.*, 2004). The only locus responsible for efficient resistance against race 1 of *V. dahliae*, *Ve1*, was isolated from tomato through map-based cloning (Kawchuk *et al.*, 2001). Fradin *et al.* (2009) have shown that EDS1, NDR1, MEK2 and SERK3/BAK1 are required for the signalling cascade downstream of Ve1 in tomato. AtEDS1, AtNDR1 and AtSERK3/BAK1 also act as positive regulators in Ve1-mediated resistance signalling in *Arabidopsis* (Fradin *et al.*, 2011), which indicates that some key components that participate in the signalling cascade downstream of *Ve1* are conserved between tomato and *Arabidopsis*. Both GhNDR1 and GhMKK2 improve cotton (*G. hirsutum*) resistance to *V. dahliae*, indicating the existence of similar defence signalling pathways among various species (Gao *et al.*, 2011). Developing an effective and practical *Verticillium* wilt control strategy is difficult at best. Genetic engineering using plant resistance genes provides an alternative to traditional crop breeding (Dubouzet *et al.*, 2011; Lacombe *et al.*, 2010; Bai *et al.*, 2011; Helliwell *et al.*, 2013; Bouwmeester *et al.*, 2014). More cotton orthologues of tomato *Ve1* have been isolated and have been suggested to confer resistance to *V. dahliae* (Gao *et al.*, 2011; Zhang *et al*., 2011, 2012). However, heterologous expression of *Ve1* in cotton did not improve tolerance to *V. dahliae* in cotton, which suggests a difference in the mechanism resistance to *V. dahliae* in cotton compared to in tomato and *Arabidopsis* (Liu *et al.*, 2014).

Research on *Arabidopsis* is pivotal to understanding molecular processes and gene regulatory networks, allowing the rapid utilization of this information into other plant species (Jansen *et al.*, 2013). A large database of manually curated and quality-controlled Affymetrix microarrays is publicly available through web-based resources (Peng *et al.*, 2007; Frickey *et al.*, 2008). With the help of Genevestigator, stress-related genes have been identified in model dicotyledonous plants (Liu *et al.*, 2008; Atkinson *et al.*, 2013). To date, 21728 unique expressed sequence tags (ESTs) are publicly available [National Center for Biotechnology Information (NCBI), www.ncbi.nih.gov], and the functions of these ESTs from cotton are largely unknown. With the completed sequencing of the cotton (*Gossypium raimondii*) genome (Wang *et al.*, 2012*b*; Paterson *et al.*, 2012) and transcriptome profiling analyses such as RNA-seq (Xu *et al.*, 2011*b*) or sequencing whole cDNA libraries (Zhang *et al.*, 2013), more efficient and practical protocols are urgently needed for high-throughput screening studies of cotton in the functional genomics era.

Using the available *Arabidopsis*-based resources, we focused on a low-cost and straightforward approach for high-throughput identification of cotton genes responsive to *V. dahliae* inoculation based on bioinformatics and genetics. Differentially expressed genes were identified in *Arabidopsis* following infection with three pathogens—*Phytophthora infestans*, *Botrytis cinerea* and *Blumeria graminis*—which have typical hemi-biotrophic, necrotrophic and biotrophic lifestyles respectively, and the genes were used to isolate homologues genes in cotton. The expression patterns of the cotton genes were evaluated through quantitative real-time PCR (qPCR). Furthermore, functional characterization of six candidate genes was performed by heterologous expression or a loss-of-function approach through virus-induced gene silencing (VIGS). The successful identification of three genes responsive to *V. dahliae* infection from six candidates suggests that the combination of a data-mining strategy and VIGS provides a new way to take full advantage of the information obtained from model plants and apply it to cotton functional genomics.

## Materials and methods

### Isolation and bioinformatic analysis of genes putatively responsive to a broad spectrum of pathogens in *Arabidopsis* and cotton

Using the web-based analysis system Genevestigator (www.genevestigator.com), three transcriptome databases of *Arabidopsis* genetrated following inoculation with *P. infestans*, *B. cinerea* and *B. graminis* were analysed. A cutoff value of 2-fold change (the absolute value of log_2_ ≥ 1) was adopted to identify genes that were differentially expressed and considered as potential candidate genes involved in the broad-spectrum response to pathogens. Gene Ontology (GO) analysis for gene information annotation and functional category distribution frequency was performed using Blast2GO software (Conesa and Götz, 2008). Kyoto Encyclopedia of Genes and Genomes (KEGG) analysis was carried out at The Arabidopsis Information Resource (TAIR; Columbus, OH, USA). The selected *Arabidopsis* candidate genes were employed as reference sequences, and the corresponding homologous genes in cotton were selected by tblastn from NCBI with a cutoff of *e* < 10^−5^.

### Expression pattern analysis of candidate genes in cotton in response to *V. dahliae* and JA

Cotton seedlings of *Gossypium barbadense* cv. 7124 were cultivated in a greenhouse at 25 °C with a 16h light/8h dark photoperiod. A highly aggressive strain of *V. dahliae*, V991, was used for pathogen inoculation in this study. The fungus was maintained on potato dextrose agar at 25 °C for 3–4 days. The conidia were washed with sterile distilled water and the spore suspension was diluted to a final concentration of 10^5^ conidia·ml^−1^. For inoculation, the seedlings were gently uprooted and rinsed in water. Roots were dipped into the spore suspension for 1min and then returned to the pots. Root samples were collected at 0, 24 and 48h after pathogen inoculation. For JA treatment the seedlings were treated similarly except that the root-dip treatment occurred in the presence of 100 µM JA. Root samples were collected at 0, 1 and 4h after hormone treatment. Seedlings inoculated with water were used for the mock pathogen infection.

The total RNA from root samples was extracted according to the protocol described by Zhu *et al.* (2005) and reverse transcribed to cDNA using 3 µg of RNA with Script III reverse transcriptase (Invitrogen, Carlsbad, CA, USA). qPCR was adopted for the expression pattern analysis of candidate genes in response to *V. dahliae* invasion or under JA treatment. The relative expression data are presented as the means ± SD from three technically independent experiments. Primer sequences of the 38 selected candidate genes are provided in Supplementary Table 1. Two-tailed Student’s *t* tests were used for statistical data analysis. Genes were considered to be differentially expressed at any time point if they showed at least a 2-fold change (log_2_ ≥ 1) upon *V. dahliae* invasion coupled with *P* < 0.001, compared with mock-treated controls.

### Functional characterization of candidate genes by VIGS

The binary TRV vectors pTRV-RNA1 and pTRV-RNA2 were used for VIGS analysis (Fradin *et al.*, 2009). The sequences of TC160375, TC141300, TC134956, TC148709 and ES802062 were amplified with the primers listed in Supplementary Table 2. The PCR fragments were inserted into pTRV-RNA2 (pYL156) and these pYL156 derivatives were transformed into *Agrobacterium tumefaciens* GV3101 by electroporation. The cultures of *A. tumefaciens*-containing vectors were prepared and infiltrated into two fully expanded cotyledons of 10-day-old seedlings as previously described by Gao *et al.* (2013). The corresponding primers in Supplementary Table 1 were used to determine the expression levels of target genes (accessions TC160375, TC141300, TC134956, TC148709 and ES802062) in the root tissue of cotton seedlings 3 weeks after VIGS treatment. Transcript levels of *GhLOX1*, *GhAOS* and *GhJAZ1* from roots of seedlings were determined by qPCR with the corresponding primers (Supplementary Table 2).

### Heterologous expression of *GhFMO1* in tobacco

The full-length cDNA sequence of *GhFMO1* was amplified through 5′ rapid amplification of cDNA ends (RACE) PCR based on the EST accession ES844424. Phylogenic analysis of the protein putatively encoded by *GhFMO1* and the homologous AtFMO was conducted using the MEGA 4.0 program. Two-week-old seedlings of *G. hirsutum* were root dipped and treated with 10mM SA similar to the method used for JA treatment. Root samples were collected 0, 4, 12 and 24h after hormone treatment. The expression pattern of *GhFMO1* under SA treatment was subsequently detected using the primer pair listed in Supplementary Table 1 for qPCR. Seedlings grown under standard conditions were used as the control.

Full-length *GhFMO1* was inserted into expression vector pGWB408 with the gene-specific primer pair FMO1-F/FMO1-R (Supplementary Table 2) through BP and LR reaction (Helliwell *et al.*, 2002). The constructed expression vector was transformed into tobacco (*Nicotiana benthamiana*) by *A. tumefaciens* EHA105 as previously described (Horsch *et al.*, 1985). Transgenic tobacco plants were selected on kanamycin and confirmed by RT-PCR using the primers rFmo1-F/rFmo1-R (Supplementary Table 2). Three single insertion lines, F-1, F-2 and F-4, which had modest, high and low expression levels respectively, were used for further study. qPCR was also performed to determine the expression levels of *NbAPX*, *NbCAT*, *NbSABP2*, *NbNPR1* and *NbPR-1* in wild-type (WT) and transgenic tobacco plants. The primer sequences for these genes are shown in Supplementary Table 2.

### qPCR and RT-PCR

For qPCR experiments, a SsoFast EvaGreen Supermix Kit (Bio-RadLaboratories, Inc., California, USA) was used following the the manufacturer’s protocol. Totally 20 µl of reaction mixture containing diluted cDNA and EvaGreen Supermix was used for qPCR (ABI Prism 7500; Applied Biosystems, Foster City, CA, USA). The qPCR amplification procedure was as follows: 94 °C for 1min, followed by 40 cycles of 94 °C for 5 s and 60 °C for 35 s. The cotton *UBQ7* (DQ116441) gene was used as the internal control gene, and relative expression levels representing the relative fold changes as compared with the expression level of *UBQ7* were calculated with 2^−△*Ct*^ (Tu *et al.*, 2007; Schmittgen and Livak, 2008). All primers for qPCR were designed by using Primer Premier 5.0 software, and were verified to produce a single peak in the melting curve using ABI Prism 7500. Dissociation parameters were a 95 °C hold for 15 s, 60 °C for 1min and 95 °C for 15 s. The RT-PCR programme was as follows: one cycle of 5min at 94 °C as an initial denaturation step followed by denaturation for 30 s at 94 °C, annealing for 30 s at 55 °C, extension for 30 s at 72 °C for 35 cycles and a final step at 72 °C for 10min.

### Pathogen inoculation and fungal recovery assay

For fungus inoculation, seedlings of *G. hirsutum* cv. YZ-1 that underwent VIGS treatment, and 3-week-old WT or transgenic tobacco plants were each gently uprooted, the roots were first rinsed in water and subsequently dipped into the spore suspension of 1×10^5^ conidia·ml^−1^ for 1min. The plants were then replanted in fresh soil to monitor disease development. The fungal recovery assay in cotton was performed according to the method of Fradin *et al.* (2009). Stem sections immediately above cotyledons were taken from cotton seedlings 10 days after *V. dahliae* inoculation and surface sterilized. The stem sections were subsequently cut into 5–8mm slices and incubated at 25 °C on potato dextrose agar. Cotton seedlings infiltrated with binary vectors pTRV-RNA1 and pTRV-RNA2 were used as the vector control, and the plants of WT and transgenic tobacco inoculated with water were used as mock controls. The disease index (DI) of *Verticillium* wilt was calculated according to the following formula:

$$\text{DI}=\frac{\sum \left(n\;\times \text{ number of leaves at level }n\right)}{\text{4 }\times \text{ the number of total leaves}}\times 100$$

Cotton leaves were classified in one of five levels of severity of disease symptoms during fungal invasion, and *n* denotes the disease level from 0 to 4 (Xu *et al.*, 2012*a*). The resistance of tobacco to *V. dahliae* was evaluated by determining the extent of stunting (leaf size, weight of the plant) as well as the degree of damage to infected leaves (leaf chlorosis/wilting). The same formula above was adopted for disease index calculations in tobacco.

*B. cinerea* was incubated on potato dextrose agar medium for 4 days and then put onto leaves in concentric circles using a punch. A fungus with identical virulence was inoculated for 72h onto the second leaf *in vitro*, collected from the top of cotton seedlings. Growth of *B. cinerea* was estimated by the size of the necrotic lesions on the leaves.

### Estimation of H_2_O_2_ production

H_2_O_2_ accumulation was determined according to the method of Gao *et al.* (2013), and visualized histochemically as a reddish-brown coloration.

## Results

### Genome-wide screening of *Arabidopsis* genes putatively involved in broad-spectrum resistance to pathogens

**Fig. 1.**
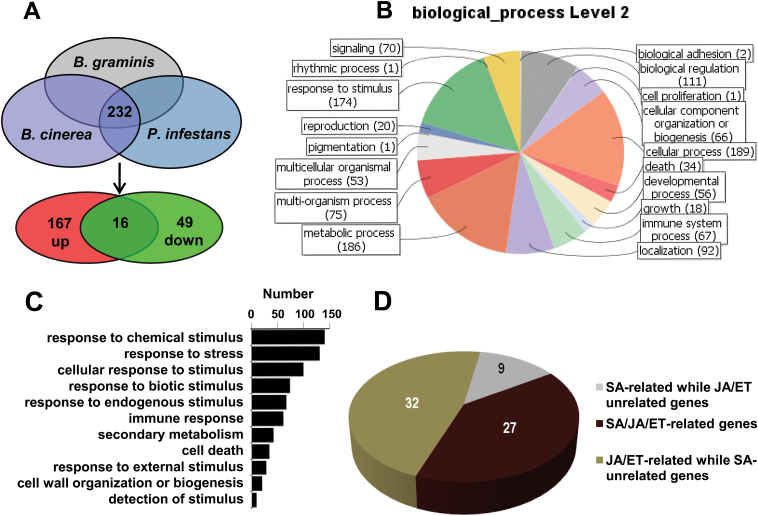
Transcriptomic screening of candidate genes in *Arabidopsis* performed by Genevestigator and GO categorization. (A) Venn diagrams showing overlap between differentially expressed genes in *Arabidopsis* in response to *B. graminis*, *B. cinerea* and *P. infestans* infection based on GeneChip. A total of 232 genes were differentially expressed upon inoculation with these three pathogens. A total of 167, 49 and 16 of these 232 genes were upregulated, downregulated and showed complex expression patterns, respectively, upon inoculation with each of these three pathogens. (B) GO categorization of 232 selected *Arabidopsis* genes at biological process level 2. (C) Partial results of 232 selected genes categorized at biological process level 3. The *y*- and *x*-axes refer to defence-related GO categories and numbers of genes, respectively. (D) Number of SA- or JA/ET-related genes among 232 *Arabidopsis* genes selected according to GO functional analysis at biological process level 6. A colour version of this figure is available at *JXB* online.

Transcriptome data from three microarray databases for *Arabidopsis* inoculated with pathogens (*P. infestans*, *B. cinerea* and *B. graminis* respectively) was analysed to screen for genes potentially involved in plant basic innate immunity. In total, 232 genes that showed differential expression levels in *Arabidopsis* upon inoculation with the three pathogens were detected (Fig. 1A, Supplementary Table 3). Among them, 167 genes were upregulated and 49 genes were downregulated in *Arabidopsis* upon inoculation with these pathogens. The remaining 16 genes showed complex expression profiles following inoculation (Fig. 1A, Supplementary Table 3).

GO categorization of the 232 genes at biological process level 2 was performed using GO functional annotations. The results showed that these genes could be divided into 18 classes (Fig. 1B), and a remarkably wide range of genes belonged to defence-related GO categories. A total of 174 genes were responsive to stimulus, 67 were involved in immune system processes and 34 associated with cell death (Fig. 1B). A more detailed analysis at biological process level 3 showed that 130 genes were responsive to stress, 141 to chemical stimulus and 73 to biotic stimulus, 42 participated in secondary metabolism and 20 were associated with cell wall organization (Fig. 1C). These data suggest the enrichment of defence-related genes among the 232 candidates. In addition, approximately 81% of the 232 genes were involved in cell processes, 80% in metabolic processes and 48% in biological regulation respectively, indicating that altered plant growth processes are detectable at the transcriptional level following fungal invasion (Fig. 1B).

The key role of hormones, especially SA and JA/ET, in the plant immune response is supported by compelling evidence. Functional analysis of the hormone-dependent genes among the 232 candidates showed that 36 and 59 transcripts were SA and JA/ET hormone-dependent respectively, of which 27 genes appeared to be involved in both hormonal signalling pathways (Fig. 1D, Supplementary Table 4). Interestingly, a large number of these hormone-related genes belong to several categories related to reactive oxygen species (ROS), MAPK cascade and SAR (hypersensitive response, programmed cell death and hydrogen peroxide metabolic process; Supplementary Fig. 1). Metabolic networks in which these 232 candidate genes might be involved were determined through KEGG analysis. Results suggested that drug metabolism catalysed by cytochrome P450 or other enzymes was a major form of defence, and the importance of flavonoid, phenylpropanoid and glucosinolate metabolism in broad-spectrum disease response was indicated (Supplementary Table 5).

### Expression patterns of candidate genes in cotton inoculated with *V. dahliae* or JA treatment

A total of 229 cotton ESTs corresponding to 232 *Arabidopsis* candidate genes were found through tblastn analysis with a ‘positives’ range from 44 to 98% (Supplementary Table 3). A comparative analysis was performed between these 229 candidates and transcriptome data obtained using RNA-seq or suppression-subtractive hybridization (or SSH) in cotton upon inoculation with *V. dahliae* in our previous work (Supplementary Table 3; Xu *et al*., 2011*a*, 2011*b*). The results showed that 84 of the 229 candidates were also detected in our previous transcriptome analysis. According to the bioinformatic analysis of the transcriptome data identified by RNA-seq or suppression subtractive hybridization, some biological processes such as ROS-related processes, ET signal cascades and secondary metabolisms were identified in the cotton defence response against *V. dahliae* (Xu *et al.*, 2011*a*, 2011*b*). Consistent with this result, the biological processes were also observed according to GO categorization of the 229 candidates in this study. In addition, the defence-related metabolic pathways catalysed by cytochrome P450, phenylpropanoid and flavonoid metabolism detected in our work were also identified from the transcriptome data in cotton or in the *Arabidopsis* response to *Verticillium* inoculation (Xu *et al.*, 2011*a*, 2011*b*; König *et al.*, 2014).

Based on GO categorization and functional annotation provided by TAIR, 38 cotton ESTs with predicted roles in defence responses were identified with at least modest homology (>50% positive) to *Arabidopsis* genes (Table 1, Supplementary Table 6). To evaluate the roles of the selected genes, their expression patterns in cotton upon *V. dahliae* infection were surveyed via qPCR. The results revealed that 24 genes were differentially regulated (≥2-fold change in gene expression with *P* < 0.0001), including 17 upregulated genes and five downregulated genes, while two genes showed complex expression patterns at all time points after inoculation with *V. dahliae* (Supplementary Fig. 2). Most of these 24 genes were also involved in ROS-related processes, hormone signal cascades or secondary metabolisms in cotton immune response. Five of these 24 genes are not currently well characterized in *Arabidopsis* or other plants (Supplementary Fig. 2, Supplementary Table 6). No significant change in expression level was found for the other 14 genes.

**Table 1:** Thirty eight genes identified as putatively responsive to a broad spectrum of fungal infection in *Arabidopsis* and the homologous ESTs from *G. hirsutum*

AGI code	Description	Accession no. of cotton EST	Identities	Positives
AT1G30700	FAD-binding domain-containing protein	TC163182	53%	68%
AT1G19250	FMO1	ES844424	39%	57%
AT2G35980	NHL10	TC139374	49%	63%
AT5G13080	WRKY75	TC143058	84%	93%
AT5G10520	RBK1	DT463342	64%	82%
AT1G07260	UGT71C3	TC169837	47%	64%
AT3G12500	PR3	TC130556^a^	69%	78%
AT2G34500	CYP710A	TC160375^a^	73%	85%
AT1G21240	WAK3	TC166725	40%	56%
AT5G40990	GLIP1	TC173925^a^	51%	67%
AT3G54640	TSA1	TC134669	70%	83%
AT1G30135	JAZ8	ES819370	42%	57%
AT5G44480	GAE1	TC170055	86%	92%
AT4G37370	CYP81D8	TC134956	64%	80%
AT5G03610	GDSL-motif lipase/hydrolase family protein	TC173850^a^	55%	69%
AT2G26560	PLA2A	TC129599^a^	67%	82%
AT3G04720	PR4	TC173903^a^	55%	65%
AT2G43820	UGT74F2	TC144828^a^	46%	66%
AT1G69490	NAP	TC170351^a^	63%	74%
AT1G09970	RLK7	TC156612^a^	71%	86%
AT1G80840	WRKY40	TC148447^a^	54%	71%
AT5G51060	RHD2	TC135821^a^	78%	87%
AT5G27600	LACS7	TC138391^a^	79%	88%
AT3G14680	CYP72A14	TC145315^a^	59%	79%
AT5G24240	PI3_PI4_ kinase	TC149040	65%	75%
AT5G49520	WRKY48	TC140811^a^	54%	65%
AT2G24180	CYP71B6	TC139031	42%	61%
AT1G22360	UGT85A2	TC139985	65%	80%
AT5G17990	TRP1	TC147379^a^	77%	86%
AT4G31970	CYP82	TC141300^a^	48%	68%
AT3G51860	CAX3	TC166651	58%	66%
AT2G37260	TTG2	TC132413	48%	59%
AT2G38120	AUX1	TC169259^a^	86%	91%
AT4G21200	GA2OX8	TC134243	51%	67%
AT4G02130	GATL6	TC153136	70%	78%
AT5G40380	RLK42	TC148709	54%	70%
AT2G42380	bZIP34	ES802062	49%	57%
AT1G78430	RIP4	TC160514	40%	50%

^a^ Genes detected by RNA-seq or suppression-subtractive hybridization method in cotton during defence response against *V. dahliae*.

**Fig. 2.**
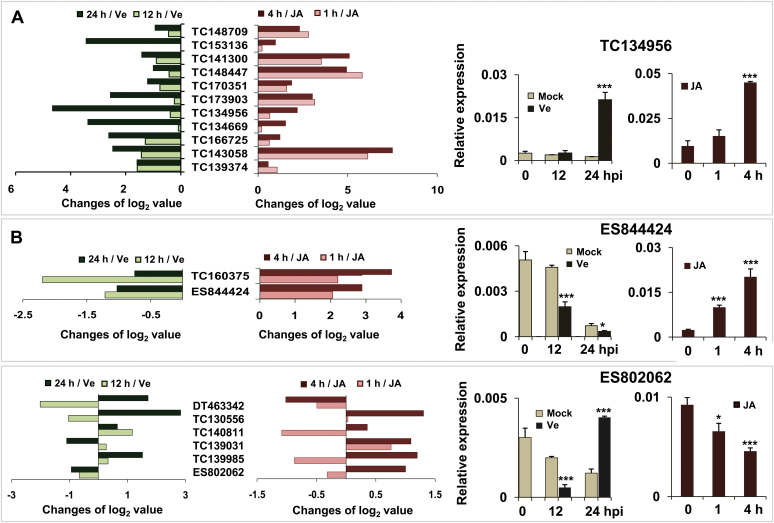
Comparison of 19 cotton gene expression profiles (log_2_ value ≥ 1; *P* < 0.0001) upon *V. dahliae* inoculation with that after JA treatment. qPCR was peformed to determine the transcript changes of 38 candidates in response to *V. dahliae* invasion or JA treatment. Nineteen of 38 genes were simultaneously and differentially regulated by pathogen infection and JA stimuli. Differentially regulated genes were clustered into three types. (A, B) Gene expression upon fungal infection showing similar (A) and opposite (B) expression patterns in response to JA treatment. (C) Genes showing complex expression patterns. Changes of log_2_ values denote the fold changes in expression level after fungal inoculation or treatment, as compared to mock-treated plants. The three types were exemplified by TC134956, ES844424 and ES802062. Each column represents three technically independent experiments. * and *** indicate signiﬁcant differences relative to the mock-treated plants at *P* < 0.05 and *P* < 0.0001, respectively. Mock, inoculated with water; Ve, inoculated with *V. dahliae*; hpi, hours post-inoculation. A colour version of this figure is available at *JXB* online.

Given that JA-dependent signal transduction was suggested to be responsible for resistance to *V. dahliae* in *Arabidopsis* and tomato (Fradin *et al.*, 2011), the expression patterns of these genes were also investigated in cotton under JA treatment. A remarkable change in the expression of 31 genes was observed, with 28 upregulated, one downregulated and two genes showing complex expression patterns (Supplementary Fig. 3). A large overlap existed between the 24 genes that exhibited obvious changes in expression upon *V. dahliae* inoculation and the 31 genes responsive to JA treatment. Approximately 79% of these genes (19 genes) were simultaneously and differentially regulated by pathogen infection and JA stimuli (Fig. 2). Of these 19 genes, 11 genes were upregulated by both pathogen inoculation and JA stimuli (Fig. 2A). Two genes, TC160375 and ES844424, were downregulated by pathogen inoculation but upregulated under JA treatment (Fig. 2B). No observed regularity was found based on expression changes in the other six genes under the two treatments (Fig. 2C).

### Comparative analysis of expression profiles of cotton genes and their homologues in *Arabidopsis*

**Fig. 3.**
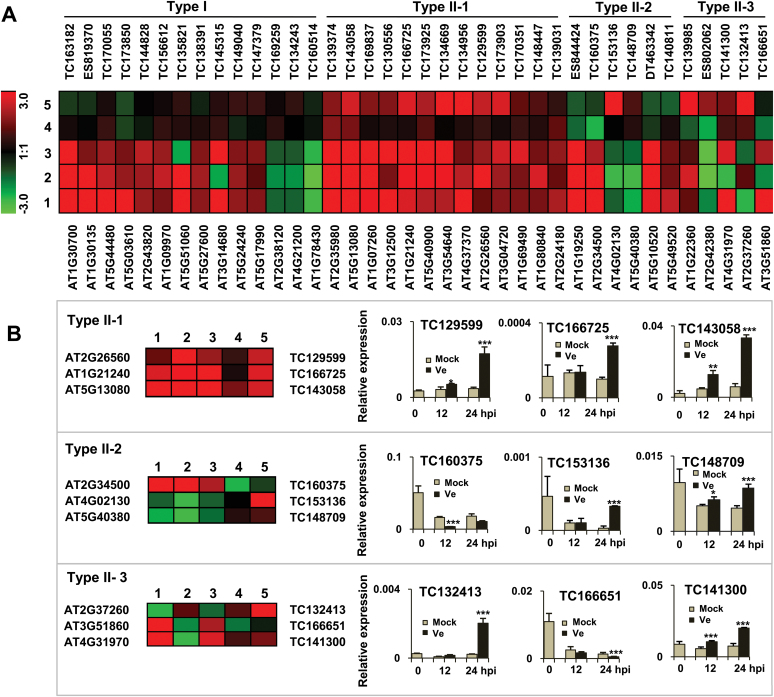
Transcriptional profiling of 38 genes in *Arabidopsis* based on GeneChip analysis using Genevestigator and the corresponding cotton ESTs based on qPCR. (A) All the genes were divided into two types based on the expression pattern of cotton genes upon *V. dahliae* inoculation. Type I refers to cotton genes with no significant changes in expression accompanied by the corresponding *Arabidopsis* genes. Type II represents differentially regulated cotton genes and with homologues in *Arabidopsis*, with 13 (II-1), six (II-2) and five genes (II-3) showing similar, opposite and complex expression patterns, respectively, in response to *V. dahliae* infection compared with the changes in the expression of the corresponding *Arabidopsis* homologues after *B. cinerea*, *B. graminis* and *P. infestans* infection, respectively. (B) Type II-1, II-2 and II-3 are exemplified with three genes belonging to each type; 1–3 represent the transcriptional changes of 38 genes in *Arabidopsis* response to *B. cinerea*, *B. graminis* and *P. infestans* infections, respectively; 4 and 5 refer to expression changes of 38 homologues in cotton at 12 and 24h post-*V. dahliae* invasion, respectively. The colour scheme represents the log_2_ ratio, with red indicating upregulation, green downregulation and black signifying no change in expression. Three technicially independent experiments were performed. ** and *** indicate signiﬁcant differences relative to the mock-treated plants at *P* < 0.01 and *P* < 0.0001, respectively. Mock, water inoculation; Ve, *V. dahliae* inoculation; hpi, hours post-inoculation. A colour version of this figure is available at *JXB* online.

Compared to the expression pattern of the 38 homologous genes in *Arabidopsis*, the expression levels of 24 genes were differentially regulated in cotton after *V. dahliae* infection (Fig. 3). No change was found in the other 14 genes in cotton (Fig. 3A, Type I). The results of the transcriptional profiling of 13 genes in cotton performed by qPCR were in agreement with those for homologous genes in *Arabidopsis* following fungal inoculation, according to GeneChip analysis (Fig. 3, Type II-1). However, completely opposite expression patterns upon infection with pathogens in cotton and *Arabidopsis* were also found for six genes. For example, the expression level of TC160375 was significantly downregulated upon infection with *V. dahliae*, while its homologue AT2G34500 was upregulated in response to multiple species of pathogens in *Arabidopsis* (Fig. 3, Type II-2). Meanwhile, five genes showed complex expression patterns in cotton and *Arabidopsis* in response to pathogens (Fig. 3, Type II-3). TC141300 transcripts were upregulated in response to *V. dahliae* in accordance with the transcriptional changes in AT4G31970 upon *B. cinerea* and *P. infestans* but in contrast to the response after inoculation with *B. graminis* (Fig. 3, Type II-3).

### Characterization of candidate genes with differential expression in cotton responsive to *V. dahliae* through VIGS

As described above, the functional categories of the 24 differentially expressed genes in response to *V. dahliae* were mainly in ROS-related defence, hormone-mediated signal cascades and secondary metabolic pathways. The functions of some candidate genes are not yet determined. To further evaluate the viability of our strategy, several candidate genes, namely ES844424, TC134956, TC160375, TC141300, TC148709 and ES802062, were selected for functional identification (Supplementary Table 6). Five candidate genes, EST TC134956 (Type II-1), TC160375 (Type II-2), TC148709 (Type II-2), TC141300 (Type II-3) and ES802062 (Type II-3), were chosen for functional identification through VIGS and effects on disease formation.

**Fig. 4.**
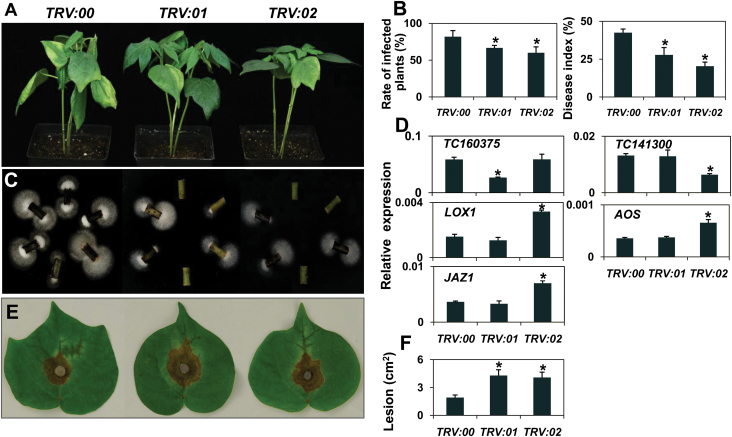
TC160375- and TC141300-VIGS cotton plants show enhanced *Verticillium* resistance but were more susceptible to *B. cinerea*. (A) Ten-day-old cotton plants were agroinfiltrated with recombinant TRV vector carrying a fragment of TC160375 or TC141300. TRV empty vector was also agroinfiltrated into plants as the vector control. Two weeks later, these plants were inoculated with *V. dahliae*. Photographs were taken 8 days after *V. dahliae* inoculation. (B) The proportion of infected plants and disease index of VIGS-silenced plants 10 days post-inoculation by *V. dahliae*. (C) For the fungal recovery assay, the stem sections taken from cotton seedlings 10 days after *V. dahliae* inoculation were incubated at 25 °C on potato dextrose agar, photographed 4 days after inoculation. (D) Transcript levels of TC160375, TC141300 and several JA-dependent genes were determined by qPCR. TC160375 and TC141300 transcripts were detected in root tissue of VIGS plants. *UBQ7* was used as an internal control. (E) Leaves of VIGS seedlings were inoculated with *B. cinerea in vitro*, and photographed after 3 days. (F) Size of lesions induced by *B. cinerea* was measured after 3 days of inoculation. *TRV:00*, *TRV:01* and *TRV:02* refer to vector controls, TC160375- and TC141300-VIGS plants, respectively. Similar results were obtained with three replicates. Each column represents an average of three biological repeats. * Indicates signiﬁcant differences relative to the vector control at *P* < 0.05. A colour version of this figure is available at *JXB* online.

Compared with vector control plants inoculated with *V. dahliae*, no obvious difference in disease response was observed in the plants when TC134956, ES802062 or TC148709 were silenced (Supplementary Fig. 4). TC160375- and TC141300-silenced seedlings exhibited more resistance to *V. dahliae*, showing less leaf chlorosis and wilting (Fig. 4A). The results are supported by the proportion of infected plants and disease index. Approximately 65% of TC160375- and 60% of TC141300-silenced seedlings showed disease symptoms 10 days post-inoculation, while 82% of infected seedlings in the vector control showed symptoms (Fig. 4B). Moreover, there was a 34% lower disease index in TC160375 and 50% lower disease index in TC141300 plants compared to the vector control (Fig. 4B). Fungal recovery assays from stem sections of inoculated cotton also illustrated the roles of TC160375 and TC141300 in cotton resistance to *V. dahliae* (Fig. 4C).

Recent evidence suggests that activated JA signal cascades can be beneficial for *Verticillium* wilt control in tomato, *Arabidopsis* and cotton (Fradin *et al.*, 2011; Gao *et al.*, 2013). Because both TC160375 and TC141300 transcripts accumulated under JA treatment (Fig. 2), the transcripts of *AOS*, *LOX1* and *JAZ1* (involved in JA biosynthesis or JA signal cascade) were analysed in vector control and VIGS plants. The results showed that these three genes were constitutively upregulated in cotton roots after silencing TC141300, while no obvious transcriptional changes were observed in T160375-silenced plants compared with those in the vector control (Fig. 4D). These results suggest that the enhanced resistance to *V. dahliae* observed in TC141300-silenced cotton is most likely at least in part due to activation of the JA signalling pathway. The roles of TC160375 and TC141300 in response to *B. cinerea* were also investigated. The results show that resistance to *B. cinerea* was compromised in both TC160375- and TC141300-silenced cotton plants, and larger necrotic areas could be observed in leaves of TC160375- as well as TC141300-silenced plants 3 days post-infection with *B. cinerea* in comparison to vector control plants (Fig. 4E and 4F).

### Overexpression of *GhFMO1* compromises tobacco resistance to *Verticillium* wilt

**Fig. 5.**
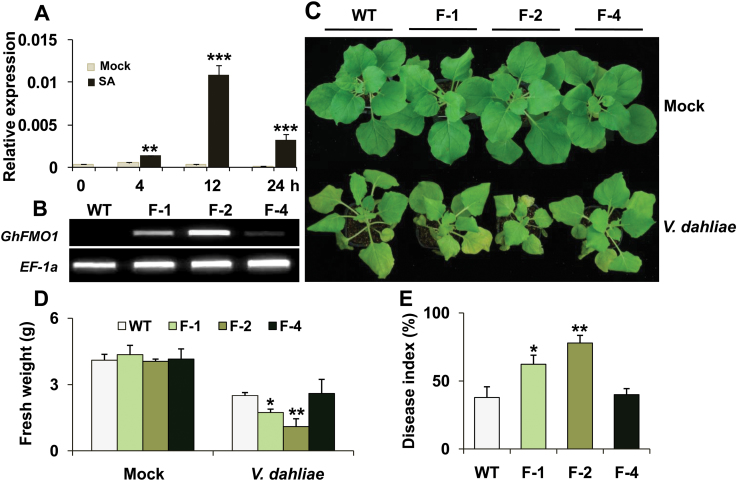
*GhFMO1* was dramatically upregulated following SA treatment in cotton and enhanced susceptibility to *V. dahliae* in tobacco. (A) Two-week-old cotton seedlings were mock-treated (control) or treated with 10mM SA. Expression of *GhFMO1* in root tissues under control conditions and SA treatment at the indicated time points was analysed by qPCR. (B) Expression analysis of *GhFMO1* in WT and transgenic tobacco lines (F-1, F-2 and F-4) by RT-PCR. *NbEF-1α* was used as an internal control in tobacco. (C) Four-week-old WT and transgenic tobacco plants were mock inoculated or inoculated with *V. dahliae*, and photographed at 14 days post-inoculation. (D, E) The weight and disease index of WT and transgenic tobacco plants were measured 14 days after *V. dahliae* infection. Each column represents the average of three independent replicates. *, ** and *** indicate signiﬁcant differences relative to the control at *P* < 0.05, *P* < 0.01 and *P* < 0.001, respectively. A colour version of this figure is available at *JXB* online.

Heterologous expression of cotton genes in tobacco was also used to investigate gene function. The full-length candidate gene with an EST identification of ES844424 (Type II-2), containing an open reading frame of 1554 nucleotides putatively encoding a peptide of 517 amino acid residues, was amplified using RACE-PCR (Supplementary Fig. 5). The protein shared 73% identity with AtFMO1 (AT1G19250) and was named GhFMO1. Phylogenic analysis shows that GhFMO1 belongs to clade I of the FMO family in *Arabidopsis*, which is involved in the plant defence response (Supplementary Fig. 6). Transcript abundance of *GhFMO1* in cotton under SA (defence-related hormone) treatment was also analysed and found increase significantly within 12h (Fig. 5A).

Three *GhFMO1*-overexpressing tobacco lines, including F-1, F-2 and F-4 with relatively moderate, high and low expression, respectively, were selected for further fungal inoculation experiments (Fig. 5B). Obvious stunting in WT and transgenic plants was observed compared to the mock-treated control 10 days post-inoculation with *V. dahliae* (Fig. 5C). However, a difference was observed between the transgenic and WT plants. The growth of the F-4 line was comparable to that of WT plants after infection with *V. dahliae*, while more severe stunting appeared in F-1 and F-2, which displayed 31 and 56% losses of fresh weight, respectively, compared with WT plants 2 weeks after inoculation with *V. dahliae* (Fig. 5D). More severe leaf wilting was found in the F-1 and F-2 lines, with a 64 and 105% higher disease index, respectively, than that in WT plants (Fig. 5E). These results suggest that expression of *GhFMO1* compromises the tobacco defence response to *V. dahliae*.

**Fig. 6.**
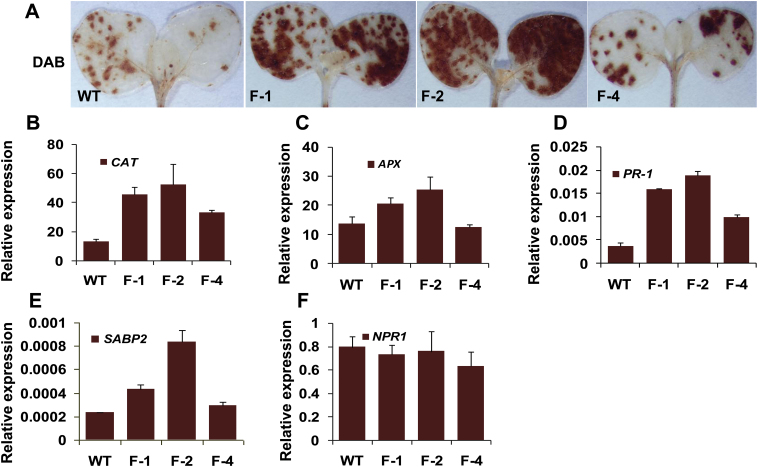
GhFMO1 causes the activation of ROS and SA signals in tobacco. (A) H_2_O_2_ production in 2-week-old WT and transgenic tobacco seedlings revealed by 3,3′-diaminobenzidine staining 12h post-infiltration. (B–F) Expression of genes involved in defence responses in WT and transgenic plants detected by qPCR. Leaf samples of 4-week-old WT and transgenic tobacco grown under standard conditions were collected for RNA extraction. Gene expression levels were normalized to *EF-1a* expression levels. A colour version of this figure is available at *JXB* online.

Previous studies have suggested a close link between ROS and FMO1 (Bartsch *et al.*, 2006). Thus, the content of H_2_O_2_ as well as transcript levels of antioxidant enzymes in WT and transgenic plants were measured. As shown in Fig. 6A, increased H_2_O_2_ accumulation was found in transgenic seedlings in a manner correlated with the transcriptional level of *GhFMO1*. Compared with WT plants, a higher expression level of two antioxidant enzymes, CAT and APX, was also detected in *GhFMO1*-overexpressing tobacco, especially in lines F-1 and F-2 (Fig. 6B and 6C). SA is one of the most important pathogen defence-related hormones. As *GhFMO1* transcripts were observed to increase dramatically after SA stimuli, the expression level of the SA-responsive marker gene *PR-1* was evaluated in the WT and transgenic lines. As expected, *PR-1* was constitutively upregulated in transgenic plants in a *GhFMO1* transcript dose-dependent manner (Fig. 6D). Therefore transgenic *GhFMO1* tobacco showed constitutively activated ROS responses accompanied by sensitized SA cascades and comprised resistance to *V. dahliae* in comparison with WT plants.

## Discussion

### High-throughput screening for *V. dahliae*-responsive genes from candidates putatively involved in basal immunity based on a data-mining strategy

*Verticillium* wilt of cotton caused by *V. dahliae* results in significant yield and fiber quality losses every year in China. Increasing the natural defences of cotton may reduce the impact of the pathogen on productivity. However, the complex genetics of cotton resistance to *V. dahliae* and our poor understanding of the molecular interaction between cotton and the fungus limits support for breeding disease-resistant varieties. As the development of sequencing technologies progresses, massive amounts of data on the transcriptional response in cotton following inoculation with *V. dahliae* have been generated (Xu *et al*., 2011*a*, 2011*b*; Zhang *et al.*, 2013), but the functional roles of only a few candidate genes have been characterized.

PTI and ETI are two perception systems that contribute to plant innate immunity (Jones and Dangl, 2006). Many believe that PTI most likely constitutes an important aspect of non-host resistance, which explains why most plants are resistant to the majority of pathogens they encounter (Zipfel *et al.*, 2004; Wan *et al.*, 2008) and that PTI could be employed in improved resistance through genetic engineering (Dubouzet *et al.*, 2011; Bouwmeester *et al.*, 2014). Although the resistance mediated by the R genes in ETI rarely confers broad-spectrum disease resistance (Jones and Dangl, 2006), the signalling pathways involved in the resistance response regulated by R genes, such as phytohormones and MAPK cascades, are somewhat conserved among plants (Pieterse *et al.*, 2009; Attaran *et al.*, 2009; Bai *et al.*, 2011; Tsuda *et al.*, 2013). All these factors potentially make it possible to isolate candidate genes that play pivotal roles in cotton based on bioinformatic analysis in *Arabidopsis*.

With the available transcriptome databases in *Arabidopsis*, a growing number of genes involved in diverse cellular processes have been explored (Zimmermann *et al.*, 2005; Mittal *et al.*, 2009; Nogueira *et al.*, 2011). In this study, using a data-mining method, 232 *Arabidopsis* candidates potentially involved in basal immune processes were selected. Bioinformatic analysis tentatively confirmed the role of these genes in broad-spectrum immunity. GO categorization revealed the enrichment of disease-responsive candidates. ROS, MAPK cascades and SAR were mainly detected among hormone-related genes by deeper functional analysis (Supplementary Fig. 1). The major roles of ROS as well as MAPK cascades in basal immunity that defend the host from broad-spectrum microbes attack have been reported (Asai *et al.*, 2002; Leto and Geiszt, 2006; Tsuda *et al.*, 2013). KEGG analysis mainly indicated changes in the metabolism of phenylpropanoids and glucosinolates and especially metabolism catalysed by cytochrome P450 or other enzymes (Supplementary Table 5), and the importance of these metabolic pathways in plant responses to a broad spectrum of microbes has already been confirmed (Dixon *et al.*, 2002; Bednarek *et al.*, 2009; Wang *et al.*, 2012*a*; Geisler *et al.*, 2013). The fact that the pathways mentioned above were reported to be regulated by JA/ET signalling pathways might provide some explanation of why more JA/ET-dependent genes were considered to be involved in broad-spectrum defence responses according to GO functional analysis (Fig. 1D) (Reymond and Farmer, 1998; Liu *et al.*, 2010; Helliwell *et al.*, 2013). Consistent with this result, most (31 of 38 genes) of the selected candidates in cotton also exhibited clear responses to JA treatment (Supplementary Fig. 3).

In total, 229 cotton ESTs, corresponding to 232 *Arabidopsis* candidate genes, were identified. Previously, 84 of 292 cotton candidates were identified by RNA-seq or suppression subtractive hybridization in cotton upon inoculation with *V. dahliae*, and some of them, such as *GbWRKY1* (TC143058), had been characterized in our previous studies (Xu *et al*., 2011a, 2011*b*, 2012*b*). Some of these 229 genes involved in ROS, hormone cascades and secondary metabolisms in cotton and *Arabidopsis* responsive to *V. dahliae* mentioned in previous reports were also detected in our study (Xu *et al*., 2011*a*, 2011*b*; König *et al.*, 2014). The expression of 24 among 38 candidates was differentially regulated by inoculation of *V. dahliae* and also predicted to be involved in ROS, hormone cascades or secondary metabolisms.

All these data indicate the high efficiency of this strategy to screen candidate genes in cotton responsive to *V. dahliae* infection. Furthermore, this strategy may help us to identify the key genes or key signal pathway. Furthermore, five of the 24 genes in response to fungus inoculation (Supplementary Fig. 2, Supplementary Table 6) were not characterized in model plants, indicating that novel genes involved in the cotton innate system may be identified by this strategy, but this requires further analysis.

Among the 229 genes, almost 50% of them showed a high degree of homology to the corresponding *Arabidopsis* gene products at the amino acid sequence level (≥80% similarity, ≥67% identity). Although more than half of the cotton genes were transcriptionally modulated the fungus (Fig. 3A), the expression profiles were largely different from those of the corresponding genes in *Arabidopsis* based on GeneChip results. The difference may be partially due to the specific mechanism of *Arabidopsis* and cotton response to pathogens or the existence of more homologous genes in allopolyploid cotton. For instance, more cotton orthologues of tomato *Ve1* have been isolated and suggested to confer resistance to *V. dahliae*, although they show much sequence diversity (Gao *et al.*, 2011; Zhang *et al*., 2011, 2012; Liu *et al.*, 2014).

### The application of VIGS or heterologous expression for characterization of candidates and the stress signalling involved in immune responses to *V. dahliae*

Functional analysis was carried out for six candidate genes using heterologous expression or an expression knock-down approach through VIGS. The results showed that *GhFMO1* overexpression aggravated *Verticillium* wilt symptoms in tobacco. SAR is a plant immune response to biotrophic pathogen attack, which is usually SA-dependent (Vlot *et al.*, 2008). It has been shown that FMO1 is a necessary component of SAR (Mishina and Zeier, 2006). Previous research reported that methylsalicylic acid (MESA, a volatile SA derivative converted from SA) is one of the metabolites involved in SAR, and *SABP2*, which encodes an esterase hydrolysing MESA to SA, is considered to be a receptor for the SAR signal (Kumar *et al.*, 2006; Maldonado *et al.*, 2002; Vlot *et al.*, 2008). In this paper we show that *SABP2* transcripts also constitutively accumulated in transgenic tobacco lines in a manner similar to the *GhFMO1* transcript dosage-dependent accumulation of *PR-1* RNA (Fig. 6E). Expression of *NPR1*, another necessary component of SAR, was also detected in *GhFMO1* overexpressors, but no obvious difference in *NPR1* at the transcript level was observed between the transgenic lines and WT plants (Fig. 6F). This may be because the constitutive level of NPR1 is sufficient to launch the subsequent signal cascades, as described previously (Van Wees *et al.*, 2000).

ROS were suggested to be crucial to SAR establishment, and enhanced ROS generation was usually associated with elevated antioxidant activity, which also plays an important role in SAR (Lee and Hwang, 2005; Leto and Geiszt, 2006). Thus, it is plausible that the priming of ROS and SA-dependent signals in transgenic plants is the cause of SAR such as when activated by GhFMO1. Gao *et al.* (2013) recently found that activation of ROS and SA signal cascades might compromise resistance to *Verticillium* wilt in *GbSSI2*-silenced cotton. Therefore, sensitized ROS- and SA-mediated responses in *GhFMO1*-expressing plants might be responsible for susceptibility to *V. dahliae*.

Two of five candidate genes were found to be responsible for altered resistance to *V. dahliae* and *B. cinerea* by VIGS. Overexpression of *CYP82C2* (AT4G31970) in *Arabidopsis* led to the significant accumulation of methyl jasmonate-induced biosynthesis of indole glucosinolates in roots even under normal conditions, and this protein might act in the metabolism of Trp-derived secondary metabolites under conditions of elevated JA levels (Liu *et al.*, 2010). Compromised resistance of the mutant to *B. cinerea* was accompanied by decreased expression of JA-induced defence genes (Liu *et al.*, 2010). Although TC141300 showed only 48% amino acid identity and 68% similarity to CYP82C2, compromised resistance to *B. cinerea* was also found when TC141300 was silenced in cotton seedlings (Fig. 4E and 4F). However, TC141300-silenced cotton seedlings showed more resistance to *V. dahliae* and activated the expression of the JA-related signalling pathway in roots (Fig. 4A–4D), in accordance with the contribution of the JA signalling pathway plant resistance to *V. dahliae* (Fradin *et al.*, 2011). The function of TC141300 needs to be further explored.

In summary, we show how a strategy of data-mining and VIGS are successfully used in combination to identify candidate genes involved in cotton response to *V. dahliae*. These results illustrate the feasibility of a data-mining approach for cotton research based on information from *Arabidopsis*. It may also be efficient for other plants with completed genome sequences but with little progress in functional genomics. Compared with a transgenic approach in functional gene characterization, VIGS can be used for high-throughput screening of target genes in a relatively short time. Together with data-mining, VIGS might be a new strategy in high-throughput screening and identification of genes involved in certain metabolic or regulatory networks in plant species.

## Supplementary Material

Supplementary material is available at *JXB* online.

Supplementary Fig. 1 GO analysis of the SA or JA/ET-related genes at biological process level 7.

Supplementary Fig. 2 qPCR analysis of 38 candidate genes in cotton upon *V. dahliae* inoculation. (A) Twenty-four genes were differentially regulated (log_2_ value ≥ 1; *P* < 0.0001); 17 genes were significantly upregulated (i), five genes were downregulated (ii) and two genes showed complex expression patterns (iii). (B) In total, 14 genes were not regulated in a clear pattern post pathogen infection. Grey and black columns refer to the relative expression levels of candidate genes after mock and pathogen inoculation, respectively. The *x*-axis represents hours post-inoculation (hpi) with *V. dahliae*. Three technical repeats were performed. *, ** and *** indicate signiﬁcant differences relative to the control at *P* < 0.05, *P* < 0.01 and *P* < 0.001, respectively.

Supplementary Fig. 3 qPCR analysis of 38 candidate genes in cotton after JA treatment. (A) Thirty one genes exhibited significant changes in expression levels (log_2_ value ≥ 1; *P* < 0.0001), with 28 genes significantly upregulated (i), one gene downregulated (ii) and two genes showing a complex expression pattern (iii). (B) Seven genes were not regulated in a clear pattern after JA treatment. The columns refer to three technical repeats of gene relative expression level at the indicated hours after JA treatment (0, 1 and 4h). *, ** and *** indicate signiﬁcant differences relative to the control at *P* < 0.05, *P* < 0.01 and *P* < 0.001, respectively.

Supplementary Fig. 4 qPCR experiments were peformed to analysis ES802062, TC148709 and TC134956 transcript abundances in root tissue of VIGS plants. *TRV:00*, *TRV:03*, *TRV:04* and *TRV:05* refer to vector control, ES802062-, TC148709- and TC134956-VIGS plants, respectively. Each column represents a mean value for three biological repeats. * Indicates signiﬁcant differences relative to the vector control at *P* < 0.05.

Supplementary Fig. 5 The full-length cDNA and predicted amino acid sequence of *GhFMO1* and its protein product.

Supplementary Fig. 6 Phylogenic analysis of the deduced amino acid sequence of *Arabidopsis* FMOs performed by the MEGA 4.0 program.

Supplementary Table 1 Primers for the 38 cotton genes used for qPCR.

Supplementary Table 2 Primers for the six candidate genes and other genes involved in defense response signalling.

Supplementary Table 3 Detailed information on 232 genes and their expression data in *Arabidopsis* challenged with *B. cinerea*, *B. graminis* and *P. infestans*, respectively, along with the corresponding cotton ESTs.

Supplementary Table 4 SA or JA/ET-dependent candidate genes identified from 232 genes in *Arabidopsis*.

Supplementary Table 5 Metabolism networks analysed by KEGG.

Supplementary Table 6 A detailed list of the 38 selected genes putatively involved in broad-spectrum immunity in *Arabidopsis* and the homologous ESTs from *G. hirsutum*.

Supplementary Data

## Abbreviations:

EST: expressed sequence tag
ET: ethylene
ETI: effector-triggered immunity
GO: Gene Ontology
JA: jasmonic acid
KEGG: Kyoto Encyclopedia of Genes and Genomes
MAPK: mitogen-activated protein kinase
PAMP: pathogen-associated molecular pattern
PTI: PAMP-triggered immunity
qPCR: quantitative real-time PCR
RACE: rapid amplification of cDNA ends
ROS: reactive oxygen species
SA: salicylic acid
SAR: systemic acquired resistance
TAIR: The Arabidopsis Information Resource
VIGS: virus-induced gene silencing
WT: wild-type.
