# Supplementary material for: Functional characterization of cotton genes responsive to Verticillium dahliae through bioinformatics and reverse genetics strategies
Source: J Exp Bot. 2014 Oct 17;65(22):6679–92. doi: 10.1093/jxb/eru393 (PMC4246195; doi:10.1093/jxb/eru393)
Supplement: Supplementary Data [file supp_65_22_6679__index.html]

Functional characterization of cotton genes responsive to Verticillium dahliae through bioinformatics and reverse genetics strategies — Functional characterization of cotton genes responsive to Verticillium dahliae through bioinformatics and reverse genetics strategies — Supplementary Data 

# Functional characterization of cotton genes responsive to *Verticillium dahliae* through bioinformatics and reverse genetics strategies

## Supplementary Data

Data files

**Files in this Data Supplement:**

- Supplementary Data - Supplementary Data
- Supplementary Data - Supplementary Data
