# Supplementary material for: Functional characterization of cotton genes responsive to Verticillium dahliae through bioinformatics and reverse genetics strategies
Source: J Exp Bot. 2014 Oct 17;65(22):6679–92. doi: 10.1093/jxb/eru393 (PMC4246195; doi:10.1093/jxb/eru393)
Supplement: Supplementary Data [file supp_eru393_jexbot130617_file001.pdf]

Functional characterization of cotton genes responsive to *Verticillium dahliae* through bioinformatics and reverse genetics strategies

Lian Xu, Wenwen Zhang, Xin He, Min Liu, Kun Zhang, Muhammad Shaban, Longqing Sun, Jiachen Zhu, Yijing Luo, Daojun Yuan, Xianlong Zhang and Longfu Zhu*

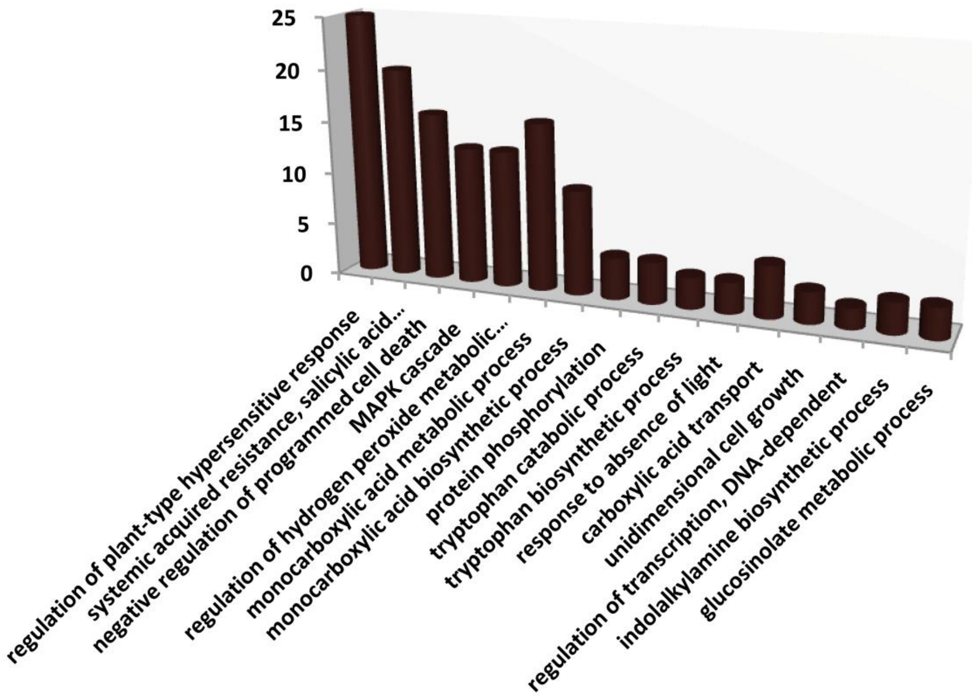

Supplemental Figure 1 GO analysis of the SA or JA/ET-related genes at biological process level 7.

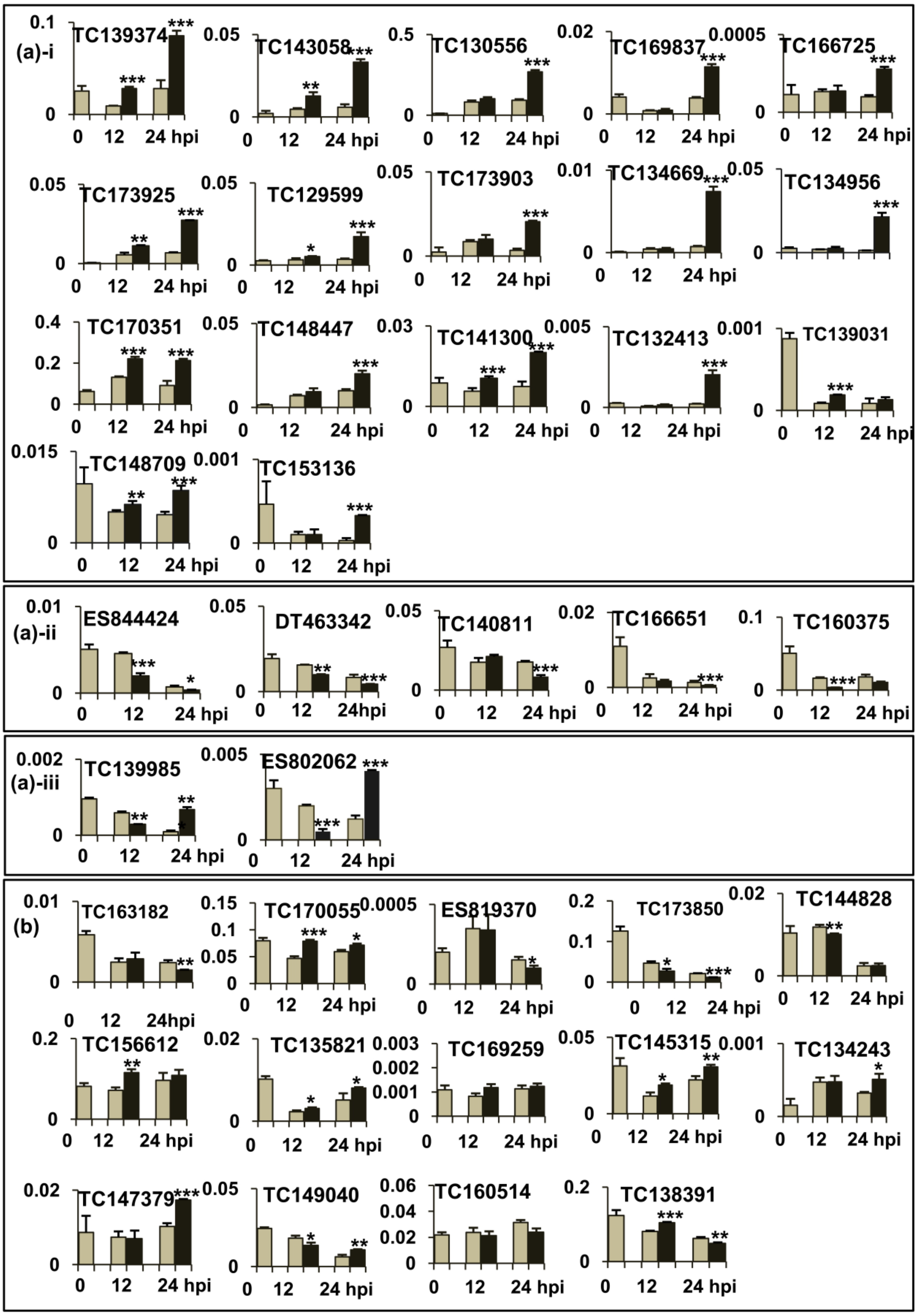

Supplemental Figure 2 qPCR analysis of 38 candidate genes in cotton upon *V. dahliae* inoculation. **(A)** Twenty-four genes were differentially regulated ($\log_2$ value ⩾ 1; P<0.0001); 17 genes were significantly upregulated (i), 5 genes were downregulated (ii), and 2 genes showed complex expression patterns (iii). **(B)** In total,

fourteen genes were not regulated in a clear pattern post pathogen infection. Grey and black columns refer to the relative expression levels of candidate genes after mock and pathogen inoculation, respectively. X-axis represents hours post inoculation (hpi) with *V. dahliae*. Three technical repeats were peformed. *, ** and *** indicate significant differences relative to the control at $P < 0.05$, $P < 0.01$, and $P < 0.001$, respectively.

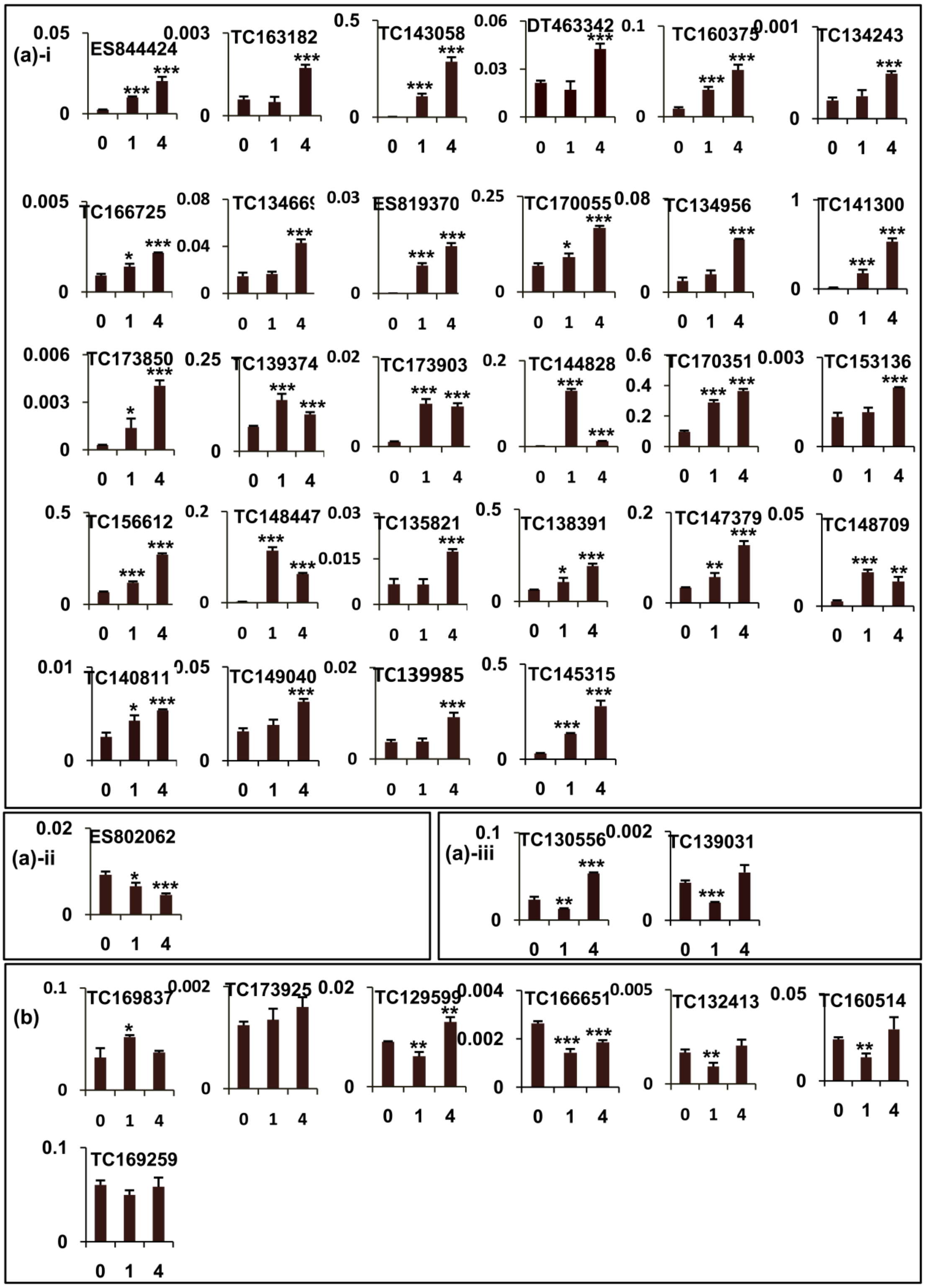

Supplemental Figure 3 qPCR analysis of 38 candidate genes in cotton after JA treatment. **(A)** Thirty-one genes exhibited significant changes in expression levels (log_2 value ⩾1; P<0.0001), with 28 genes significantly upregulated (i), 1 gene downregulated (iii) and 2 genes showing a complex expression pattern (ii). **(B)** Seven

genes were not regulated in a clear pattern after JA treatment. The columns refer to three technical repeats of gene relative expression leve at indicated hours after JA treatment (0, 1 and 4 h). *, ** and *** indicate significant differences relative to the control at $P < 0.05$, $P < 0.01$, and $P < 0.001$, respectively.

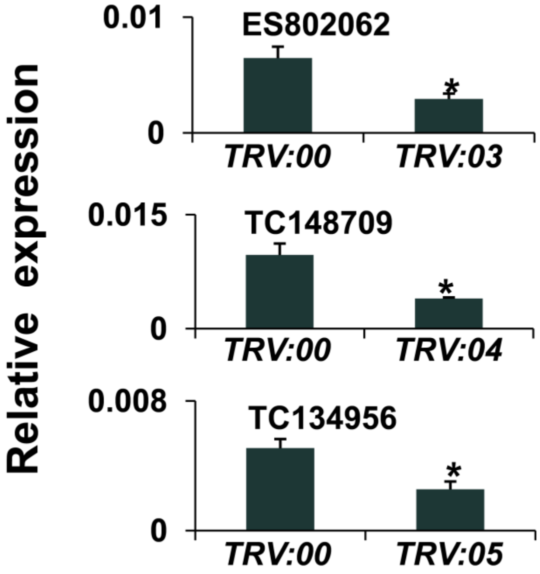

Supplemental Figure 4 qPCR experiments were peformed to analysis ES802062, TC148709 and TC134956 transcript abundances in root tissue of VIGS plants. *TRV:00*, *TRV:03*, *TRV:04* and *TRV:05* refer to vector control, ES802062, TC148709 and TC134956-VIGS plants, respectively. Each column represents a mean value for three biological repeats. * indicates significant differences relative to the vector control at $P < 0.05$.

.

gaaaacgagaacatatatcaactaaagatagca

atggacagaaaggtggccattattggtgctggcataagtggccttcttgcttgcaaatacacattgtca

M D R K V A I I G A G I S G L L A C K Y T L S

aagggttttcatccaatagtttttgagtcccaaagcactgtgggaggtgtttggaccaaaaccgtggag

K[illegible] G F H P I V F E S Q S T V G G V W T K T V E

accacaaagctccaaacacctaaaccagtttatcaattctccgatttcccatggccggagtcggtctcg

T T K L Q T P K P V Y Q F S D F P W P E S V S

gaagattttccggacggacagaaggtatttgattatgttaatgggtatgcaaagcattttgatttggtt

E D F P D G Q K V F D Y V N G Y A K H F D L V

aagcacatcaaattcaataccaaagttgttggtattgaatttgaaggtcctaatgatgaagagatccaa

K H I K F N T K V V G I E F E G P N D E E I Q

gcttggagtttatggggttgtaatggtgagccttttgggtctaaagggaaatggaaggttgttgttgaa

A W S L W G C N G E P F G S K G K W K V V V E

gatttggtaactctttccactgagatttacaatgtggactttgtaatcgtttgtgtggggagattcagt

D L V T L S T E I Y N V D F V I V C V G R F S

ggtttgccaaacatcccagaatttcccccgaaaaaaggcccggaagcatttgatggtaaggtaatacat

G L P N I P E F P P K K G P E A F D G K V I H

tcaatggaatatgctgccatggatcataaacaggctgctgaattcgtcaaaggcaaacgagtcattgtt

S M E Y A A M D H K Q A A E F V K G K R V I V

gttgggtttcaaaaatctgcactcgacattgcagtggagtgttctgctgctaatggaaaggaagatcca

V G F Q K S A L D I A V E C S A A N G K E D P

tgcacagttttatacaggacagctcactggaatgtccctgactatcttccatggggattttcacttgca

C T V L Y R T A H W N V P D Y L P W G F S L A

cacatgtaccttagccgtttctccgagcttatggttcataagcctggtgaagggcttctcctttccctc

H M Y L S R F S E L M V H K P G E G L L L S L

ttagccacaattctcacaccgctgagatatgcatattcaaaatttgttgaaagtgatattaaaaagaag

L A T I L T P L R Y A Y S K F V E S D I K K K

cttcgtttggaaaaacatgggatggtcccaacacatagcttcctcaaagaaatcagctcatgtttgatc

L R L E K H G M V P T H S F L K E I S S C L I

tcaactgtccctgaaaaattctatgacaaggttgaaaatggagaaatcaaattgaaaaaggccccaagc

S T V P E K F Y D K V E N G E I K L K K A P S

tttagtttctgcaacaatggcgttttggtcgaaggcgagacctcaccgatagaagcggaccttgtcatc

F S F C N N G V L V E G E T S P I E A D L V I

ttggccactggattcaagggtgaaaaaaagctcaaacacatttttatgtctcgaaccttccaagactac

L A T G F K G E K K L K H I F M S R T F Q D Y

attactggatcccctgatgcagcccttccactctacagggaatgcattcaaccaaggataccacaacta

I T G S P D A A L P L Y R E C I Q P R I P Q L

gcagtaatcggattctcagagagtatttcaaatatatttacctcagagatgagatgtagatgggtagca

A V I G F S E S I S N I F T S E M R C R W V A

gagcttcttgacggcacgttcaagctgccaagcattaaagacatggaaataaatataaaagaatgggat

E L L D G T F K L P S I K D M E I N I K E W D

gaatacttgaaacaatactcgggtggatactatcgtagaaagtgcattggtgctttgcatatatggtac

E Y L K Q Y S G G Y Y R R K C I G A L H I W Y

aatgatcaactgtgcaaagatatggcatggaaaccaaggagaaagaaagggtttttttgcagaactttt

N D Q L C K D M A W K P R R K K G F F A E L F

gaaccttatggtcccatggattatgtctctaattaa

E P Y G P M D Y V S N *

Gctggttcttacctttgcattgtacacaatccaaattcatcatctttattgtgtggtttgt

cccttattttcaataaatgggtttcgggt tagctacaagatactcaaaccatgtatgaag

gaagcacaatgtaagaatgttggggtctcttattgtgtgcaataataaaaaaaaaaaaaaaaaaa

Supplemental Figure 5 The full-length cDNA and predicted amino acid sequence of *GhFMO1* and its protein product.

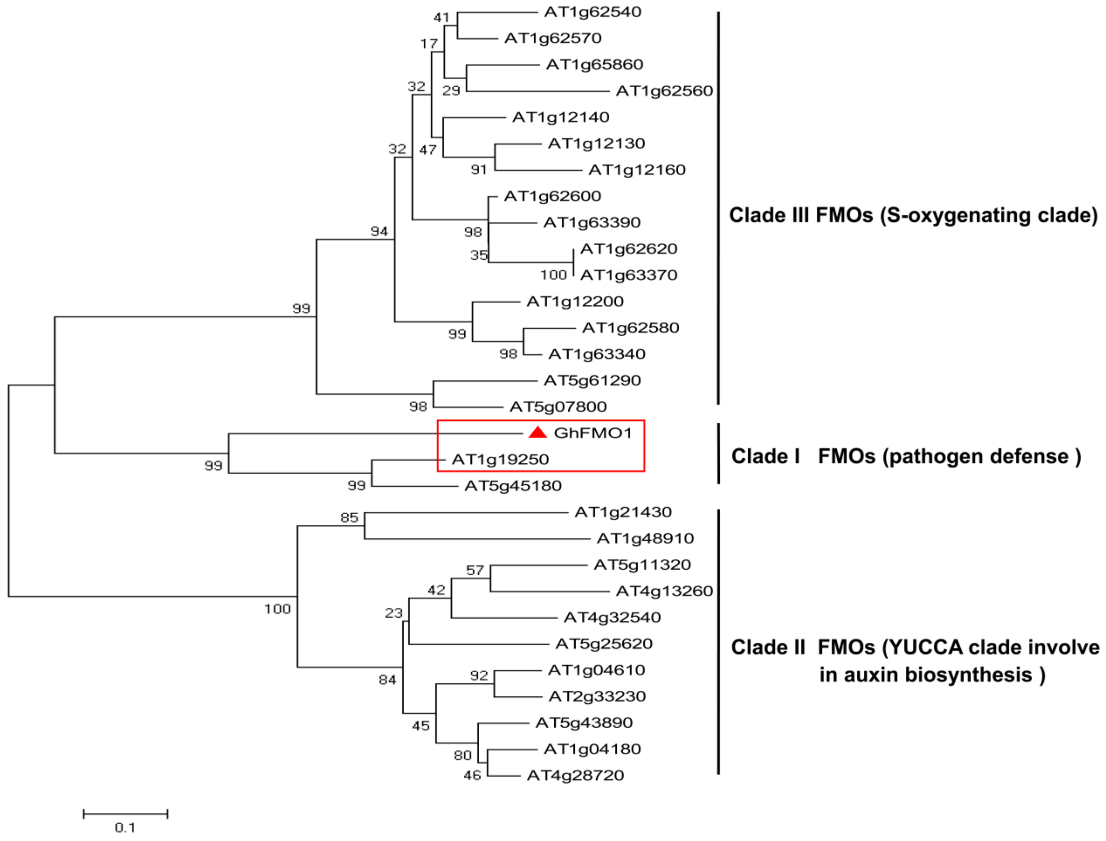

Supplemental Figure 6 Phylogenic analysis of the deduced amino acid sequence of *Arabidopsis* FMOs performed by the MEGA 4.0 program.

Supplemental Table 1 Primers of 38 cotton genes used for qPCR.

EST	Sense primer	Reverse primer
TC163182	TGTCCTACTGTTGGCGTTGGTGGTC	TTTAGCATCCACGACATGGTCCACC
ES844424	AGACCTCACCGATAGAAGCGGACCT	CCTGTAGAGTGGAAGGGCTGCATCA
TC139374	TTCATCTTTTGGCTCATCTTCCG	GCAAAGTGTTGTTGGAAGTGAGGTT
TC143058	CCACTGGACTTTGTTTTGATCCGTAG	AGGTGCCATGTTTAACGGTAATGATTG
DT463342	TCGTTCTGTTGTAGGAGCGGTTT	AAGATTGATGTATTCGCATTTGGAG
TC169837	TCAGTGGTTTTTCTTTGCTTCGG	GCAGCGTCATTTAGTGGTGTTTCT
TC130556	GATGACTCCACAATCACCGAAGC	ACACCATACCCTGTAGCCCGAC
TC160375	GCTCTCTGTGATTATTGTCCTTTGC	CAAAGAATCATAGCCACAAACAAGG
TC166725	CATTGATTCGGAACATAACTCTGGA	ACATCCATTGGGAAGGTAAGGG
TC173925	GGCAAAGCTGATGAGAAAGGAAC	GAGGAGGACTGAAATACGGTGGA
TC134669	ACAATACCCATTCTCCATCTCGCT	ATGCAACTTTGCCTTGATTTTTCA
ES819370	ACTTGGAACTTCGTCTTCTTCCTTCT	ATTGTTAGCTGTTGCTGCTGGGT
TC170055	GCTCGTGGTATTATGCCTGGTCT	CTTTACTGGGTTGTGCCTTTTGAA
TC134956	TATCATCTTCGCAAACCGACCCA	TCAACCGCCTGGTTTCGTCTTT
TC173850	GCGTTTGGAGATTCTTATGCTGATAC	AACCCATCAGAGAAACGACCAGAAG
TC129599	GACATCAAGCAACTCCAGCCAAG	TGGGCAGGAAGGTAAGTAGGAGC
TC173903	GGAGGTGCCTGAGGGTAAGAAACT	CCTACTCCATCAGTGTCCAATCGG
TC144828	GTTGTGAGGCGAGAGGAACTGAT	CCATCAGATGTTGGACGAACTTATT
TC170351	GGTGTCAGGGTATTGGAAGGCT	TCAGTCTTGAGTCCCTTTGGTGG
TC156612	AGATTTCGGACTTGCCAAGATTGT	CACATCGCTCTTCTCGTTTACTTTG
TC148447	GGCAAAAAGTCACCAGGGATAAT	TTGAGAAGGGGGAAGATGATTATG
TC135821	CTAACATCCCAGATTTCCCAAGAGT	CCACCAACAAAACCACTTCGTATT
TC138391	TTGGCACAAGGCGAATACATAGCA	TCGTACTGGATACCCTCAGATGCG
TC145315	TAAAGCAGGGGAAGAGAGCAAT	TAGTCCACACCAGTAAGACCGAAG
TC149040	CAGATGTTTGTGGAGAACCGTGG	TGTTTGCCCATCTTTATCTTTGCTC
TC140811	CAAGAGAAACCACGATTCAGTAACC	TTCTTCCTTTGCTTCTGTCCTCAT
TC139031	CCCCTTACGGTGCTTACTGGAGA	CGACAAAGATTATCGTTGGCGTAG
TC139985	TTCCGCCATTATCGTAAACACCT	GTTGAGGAGCAAGTGAAGGGGT
TC147379	AAGGTAGAAGGGTTAAATGATGCTG	CAATACATCAGCACTTCCACAGG
TC141300	TGTAGCCAAGGAAATCTTCACTGTC	GCCAGAATTGTCGGTAAGGAGAGA
TC166651	AACTCAGGAACTAAAGCAGCCATCG	TCAACTTTCATTTGTCAGCTTTCCG
TC132413	CTATCAATGCCTCACCCCCTAATGC	CGGTAGGCTTGACATCGGATTTCG
TC169259	CATTCCTTCCTTTCACAACTACCGT	TTTCCACAGTCACAGCATGTCCAC
TC134243	TGTTTCCTTCGCCTGAATCACTACC	GACGGTCACCCATTTGGAGTCTTTC
TC153136	ATTGCCACGCCAACTTCACAAACTATT	TGTGTACCCGACCCGTCTCCATTTA
TC148709	AAACCTGGTAAGATTGTTGGGATG	CTCTGTGAATAATTTTGGTCTCGGA
ES802062	TGAAGTATCAGCATTATCACCGAGG	AAATTTTATCTTGAGCCAAAGCAGC
TC160514	GGAAGAACAAAAGGGGGCACTG	CCGACATACCCAGCGAATCCAC
UB7	GAAGGCATTCCACCTGACCAAC	CTTGACCTTCTTCTTCTTGTGCTTG

Supplemental Table 2 Primers for the 6 candidate genes and other genes involved in defense response signaling.

Primers used for VIGS experiments.Sequences in red refer to restristion sites of BamHI linked to sense primers, and KpnI linked to reverse peimers

EST /Gene	Sense primer	Reverse primer
Primers for gene fragment amplification		
TC160375	CGGGGTACCAGTGGCGCGTGAGGTTAT	CGCGGATCCGAGAGAACTTTGGAG GGCTT
TC141300	CGGGGTACCTTTTTGCCTCGGAAATAGAA	CGCGGATCCAAGTGGCTTTGTTGAT GGTG
TC134956	CGGGGTACCTTCACCTCCTAAAACCACCC	CGCGGATCCTTCCCGTTCCACTGTT CTTA
TC148709	CGGGGTACCAATAAGCAGTGTGGAGCAT AAAAAC	CGCGGATCCCATAAATCTCCTCCATTG TCCCT
ES802062	CGGGGTACCAGCCTTCCTTGAACAACCAT	CGCGGATCCCCCTCGGTGATAATGC TGA
Primers for RT-PCR analysis		
TC160375	GTAGAGTTCGGGAGGAAGTATCAAG	AATACATGATTTCATCACAGCCCTC
TC141300	CGTTATTATGAGGACGGTTGCTG	TATGGGTGTCTAGTTCTTCTTGAGC
TC134956	GGGTGAAGTTTAGGCGTGATG	GCAGCATCAGAGGATACGACG
TC148709	TTTTCTATAACAATCGACACCG	TGGCAGTGCTGATGTGACTC
ES802062	GTTGTAAAACGGGCGGCCCAGTGAA	CAACAACCGCCTCCGTCATCTTCGG
LOX1	GATGGACTGGAGATTTGGTTTGC	GGAGTTCTTTCCACCAGGCTTGA
AOS	CGATGTCCCAACTTCAATCTCAAAC	ATCCGACGGTGGAGAATAAACAGT
JAZ1	GACCAAATCTTGTGGCATCTACCTC	CTTTGGTTCCACTGCTGCTGATT
BUQ7	GAAGGCATTCCACCTGACCAAC	CTTGACCTTCTTCTTCTTGTGCTTG

Primers used in *GhFMO1* overexpressed transgenic tobacco.

Primer name	sequence
FMO1-F	GGGGACAAGTTTGTACAAAAAAGCAGGCTGCATGGACAGAAAGGTGGCC AT
FMO1-R	GGGGACCACTTTGTACAAGAAAGCTGGGTCATTAGAGACATAATCCATGG GACC
rFmo1-F	TGGTGAGCCTTTTGGGTCT
rFmo1-R	ACGGCTAAGGTACATGTGTGC
APX-F	AGGACGGTTGCCCGATGCTAAA
APX-R	GATGTGCCCTTCCCAGCGTATGAC
CAT-F	CTCGTGGTTTTGCTGTCAAGTTCT
CAT-R	TTCTCCTGGATATGGGATTTAGGA
SABP2-F	TAGTGGGGCATAGTCTTGGTGGT
SABP2-R	GGAGTTGTGAACAGAATCAGGCA
PR-1F	GGTGTAGAACCTTTGACCTGGGAC
PR-1R	CTCAGCTAGGTTTTCGCCGTATTG
NPR1-1F	ATAAAAGAGGAGCACTTGAATCGG
NPR1-1R	TTCTTCAGTTGACGCTCTTCTGCC

EF-1aF	CTCCAAGGCTAGGTATGATG
EF-1aR	CTTCGTGGTTGCATCTCAAC

Supplemental Table 6 A detailed list of the 38 selected genes putatively involved in broad-spectrum immunity in *Arabidopsis* and the homologous ESTs from *G.hirsutum*.

AGI code	Description	Functions	References	Acession of Cotton EST
AT1G30700	FAD-bindin	n	-	TC163182
AT1G19250	FMO1	Cell death; Required for systemic acquired resistance	(Bartsch, *et al*., 2006; Mishina and Zeier, 2006)	ES844424
AT2G35980	NHL10	Independent of SA/JA/ET during CMV-induced HR treatment	(Zheng *et al*., 2004)	TC139374
AT5G13080	WRKY75	oxidative burst; mainly active JA/ET pathway	(Chen *et al*., 2013)	TC143058
AT5G10520	RBK1	Plant-microbe interaction	(Molendijk *et al*., 2008)	DT463342
AT1G07260	UGT71C3	Glucosylation of flavonoids	(Terasaka *et al*., 2012)	TC169837
AT3G12500	PR3	JA-inducible genes	(Thomma *et al*., 1998)	TC130556
AT2G34500	CYP710A	Biosynthesis of stigmasterol	(Griebel and rgen Zeier, 2010)	TC160375
AT1G21240	WAK3	n	-	TC166725
AT5G40990	GLIP1	Functions independently of SA but requires ET signaling	(Oh *et al*., 2005)	TC173925
AT3G54640	TSA1	The penultimate reaction in the biosynthesis of tryptophan	(Radwanski *et al*., 1995)	TC134669
AT1G30135	JAZ8	Negative regulation of JA signaling	(Chini *et al*., 2007)	ES819370
AT5G44480	GAE1	Biosynthesis of d-Galacturonate	(Seifert *et al*., 2002)	TC170055
AT4G37370	CYP81D8	Flavanoid biosynthesis	(klok *et al*. 2002)	TC134956
AT5G03610	GDSL-moti	n	-	TC173850
AT2G26560	PLA2A	scavenging and protection against ROS production	(Licausi *et al*., 2010)	TC129599
AT3G04720	PR4	JA-mediated defense system	(Thomma *et al*., 1998)	TC173903
AT2G43820	UGT74F2	Involved in metabolites of SA/tryptophan pathway	(Dean and Delaney, 2008)	TC144828
AT1G69490	NAP	responsible for senescence	(Guo and Gan, 2006)	TC170351
AT1G09970	RLK7	required for proper germination speed and tolerance to oxidative	(Pitorre *et al*., 2010)	TC156612
AT1G80840	WRKY40	Involved in SA/JA signaling	(Xu *et al*., 2006)	TC148447
AT5G51060	RHD2	Involved in antioxidant metabolism and oxidative burst	(Qureshi *et al*., 2013)	TC135821
AT5G27600	LACS7	fatty acid catabolism	(Fulda *et al*., 2002)	TC138391
AT3G14680	CYP72A14	n	-	TC145315
AT5G24240	PI3_PI4_ kinase	n	-	TC149040
AT5G49520	WRKY48	negative regulator of JA-responsive defense gene	(Xing *et al*., 2008)	TC140811
AT2G24180	CYP71B6	n	-	TC139031
AT1G22360	UGT85A2	n	-	TC139985
AT5G17990	TRP1	Tryptophan biosynthesis	(Last *et al*., 1991)	TC147379
AT4G31970	CYP82	Indole glucosinolate biosynthesis	(Liu *et al*., 2010)	TC141300
AT3G51860	CAX3	Calcium transport	(Manohar *et al*., 2011)	TC166651
AT2G37260	TTG2	Regulation of flavonoid biosynthesis	(Xu *et al*., 2013)	TC132413
AT2G38120	AUX1	Auxin polar transport/ response to IAA, ET stimulus	(Pickett *et al*., 1990)	TC169259
AT4G21200	GA2OX8	gibberellin metabolism oxidation-reduction process	(Schomburg *et al*., 2003)	TC134243
AT4G02130	GATL6	Involved in cell wall biosynthesis	(Kong *et al*., 2011)	TC153136
AT5G40380	RLK42	n	-	TC148709
AT2G42380	bZIP34	n	-	ES802062
AT1G78430	RIP4	n	-	TC160514

n, annotation concerned with biotic stress was not found.

Supplementary References

Chen X, Liu J, Lin G, Wang A, Wang Z and Lu G. 2013. Overexpression of *AtWRKY28* and *AtWRKY75* in *Arabidopsis* enhances resistance to oxalic acid and *Sclerotinia sclerotiorum*. *Plant Cell Reports* **32**, 1589-1599.

Chini A, Fonseca S, Fernández G, *et al.* 2007. The JAZ family of repressors is the missing link in jasmonate signalling. *Nature* **448**, 666-671.

Dean JV and Delaney SP. 2008. Metabolism of salicylic acid in wild-type, *ugt74f1* and *ugt74f2* glucosyltransferase mutants of *Arabidopsis thaliana*. *Physiologia Plantarum* **132**, 417-425.

Fulda M, Shockey J, Werber M, Wolter FP and Heinz E. 2002. Two long-chain acyl-CoA synthetases from Arabidopsis thaliana involved in peroxisomal fatty acid β-oxidation. *Plant Journal* **32**, 93-103.

Griebel T and Rgen Zeier J. 2010. A role for beta-sitosterol to stigmasterol conversion in plant-pathogen interactions. *Plant Journal* **63**, 254-268.

Guo Y and Gan S. 2006. AtNAP, a NAC family transcription factor, has an important role in leaf senescence. *Plant Journal* **46**, 601-612.

Klok EJ, Wilson IW, Wilson D, Chapman SC, Ewing RM, Somerville SC, Peacock WJ, Dolferus R and Dennis ES. 2002. Expression profile analysis of the low-oxygen response in Arabidopsis root cultures. *Plant Cell* **14**, 2481-2494.

Kong Y, Zhou G, Yin Y, Xu Y, Pattathil S and Hahn MG. 2011. Molecular analysis of a family of Arabidopsis genes related to galacturonosyltransferases. *Plant Physiology* **155**, 1791-1805.

Last RL, Bissinger PH, Mahoney DJ, Radwanski ER and Fink GR. 1991. Tryptophan mutants in Arabidopsis: the consequences of duplicated tryptophan synthase beta genes. *Plant Cell* **3**, 345-358.

Licausi F, Van Dongen JT, Giuntoli B, Novi G, Santaniello A, Geigenberger P and Perata P. 2010. *HRE1* and *HRE2*, two hypoxia-inducible ethylene response factors, affect anaerobic responses in *Arabidopsis thaliana*. *Plant Journal* **62**, 302-315.

Manohar M, Shigaki T, Mei H, Park S, Marshall J, Aguilar J and Hirschi KD.

2011. Characterization of *Arabidopsis* Ca^{2+}/H^{+} exchanger CAX3. *Biochemistry* **50**, 6189-6195.

Molendijk AJ, Ruperti B, Singh MK, *et al.* 2008. A cysteine-rich receptor-like kinase NCRK and a pathogen-induced protein kinase RBK1 are Rop GTPase interactors. *Plant Journal* **53**, 909-923.

Oh IS, Park AR, Bae MS, Kwon SJ, Kim YS, Lee JE, Kang NY, Lee S, Cheong H and Park OK. 2005. Secretome analysis reveals an Arabidopsis lipase involved in defense against Alternaria brassicicola. *Plant Cell* **17**, 2832-2847.

Pickett FB, Wilson AK and Estelle M. 1990. The *aux1* mutation of *Arabidopsis* confers both auxin and ethylene resistance. *Plant Physiology* **94**, 1462-1466.

Pitorre D, Llauro C, Jobet E, Guilleminot J, Brizard JP, Delseny M and Lasserre E. 2010. RLK7, a leucine-rich repeat receptor-like kinase, is required for proper germination speed and tolerance to oxidative stress in *Arabidopsis thaliana*. *Planta* **232**, 1339-1353.

Qureshi MK, Sujeeth N, Gechev TS and Hille J. 2013. The zinc finger protein ZAT11 modulates paraquat-induced programmed cell death in *Arabidopsis thaliana*. *Acta Physiologiae Plantarum* **35**, 1863-1871.

Radwanski ER, Zhao J and Last RL. 1995. *Arabidopsis thaliana* tryptophan synthase alpha: gene cloning, expression, and subunit interaction. *MGG* **248**, 657-667.

Schomburg FM, Bizzell CM, Lee DJ, Zeevaart JA and Amasino RM. 2003. Overexpression of a novel class of gibberellin 2-oxidases decreases gibberellin levels and creates dwarf plants. *Plant Cell* **15**, 151-163.

Seifert GJ, Barber C, Wells B, Dolan L and Roberts K. 2002. Galactose biosynthesis in *Arabidopsis*: genetic evidence for substrate channeling from UDP-D-Galactose into cell wall polymers. *Current Biology* **12**, 1840-1845.

Terasaka K, Mizutani Y, Nagatsu A and Mizukami H. 2012. In situ UDP-glucose regeneration unravels diverse functions of plant secondary product glycosyltransferases. *FEBS letters* **586**, 4344-4350.

Thomma BP, Eggermont K, Penninckx IA, Mauch-Mani B, Vogelsang R, Cammue BP and Broekaert WF. 1998. Separate jasmonate-dependent and

salicylate-dependent defense-response pathways in *Arabidopsis* are essential for resistance to distinct microbial pathogens. *Proceedings of the National Academy of Sciences of the United States of America* **95**, 15107-15111.

Xing DH, Lai ZB, Zheng ZY, Vinod K, Fan B-F and Chen ZX. 2008. Stress-and pathogen-induced *Arabidopsis* WRKY48 is a transcriptional activator that represses plant basal defense. *Molecular Plant* **1**, 459-470.

Xu X, Chen C, Fan B and Chen Z. 2006. Physical and functional interactions between pathogen-induced *Arabidopsis* WRKY18, WRKY40 and WRKY60 transcription factors. *Plant Cell* **18**, 1310-1326.

Xu W, Grain D, Gourrierec J, Harscoët E, Berger A, Jauvion V, Scagnelli A, Berger N, Bidzinski P and Kelemen Z. 2013. Regulation of flavonoid biosynthesis involves an unexpected complex transcriptional regulation of TT8 expression, in Arabidopsis. *New Phytologist* **198**, 59-70.

Zheng MS, Takahashi H, Miyazaki A, Hamamoto H, Shah J, Yamaguchi I and Kusano T. 2004. Up-regulation of *Arabidopsis thaliana* NHL10 in the hypersensitive response to Cucumber mosaic virus infection and in senescing leaves is controlled by signalling pathways that differ in salicylate involvement. *Planta* **218**, 740-750.
